# A Systematic Review of Cognition in Cervical Dystonia

**DOI:** 10.1007/s11065-022-09558-z

**Published:** 2023-01-25

**Authors:** Sarah O’Connor, David Hevey, Tom Burke, Shameer Rafee, Niall Pender, Fiadhnait O’Keeffe

**Affiliations:** 1 Department of Clinical Psychology, School of Psychology, Aras an Phiarsaigh, Trinity College Dublin, Dublin 2, Dublin, Ireland; 2 Department of Psychology, National University of Ireland Galway, Galway, Ireland; 3 Department of Neurology, St Vincent’s University Hospital, Dublin, Ireland; 4 School of Medicine & Health Science, University College Dublin, Dublin, Ireland; 5 Academic Unit of Neurology, Trinity College Dublin, Dublin, Ireland; 6 Department of Psychology, Royal College of Surgeons in Ireland, Dublin, Ireland; 7 Department of Psychology, St Vincent’s University Hospital, Dublin, Ireland; 8 School of Psychology, University College Dublin, Dublin, Ireland

**Keywords:** cervical dystonia, cognition, neuropsychology, systematic review

## Abstract

Growing evidence points to a spectrum of non-motor symptoms, including cognitive difficulties that have a greater impact on functional outcomes and quality of life than motor symptoms in cervical dystonia (CD). Some cognitive impairments have been reported; however, findings are inconsistent, and described across mixed groups of dystonia. The current review aimed to examine the evidence for cognitive impairments in CD. MEDLINE, EMBASE, PsychINFO and Web of Science databases were searched. Studies were included if they met the following criteria (i) cross-sectional or longitudinal studies of adults with CD, (ii) where the results of standardised measures of cognitive or neuropsychological function in any form were assessed and reported, (iii) results compared to a control group or normative data, and (iv) were published in English. Results are presented in a narrative synthesis. Twenty studies were included. Subtle difficulties with general intellectual functioning, processing speed, verbal memory, visual memory, visuospatial function, executive function, and social cognition were identified while language, and attention and working memory appear to be relatively spared. Several methodological limitations were identified that should be considered when interpreting the evidence to describe a specific profile of cognitive impairment in CD. Clinical and research implications are discussed.

**Supplementary Information:**

The online version contains supplementary material available at 10.1007/s11065-022-09558-z.

## A Systematic Review of Cognition in Cervical Dystonia

Dystonia is a movement disorder, characterised by sustained or intermittent muscle contractions that cause repetitive abnormal movements and postures (Albanese et al., 2013). It is the third most common movement disorder, after idiopathic Parkinson’s Disease and Essential Tremor (Defazio, 2010). Dystonia is classified along two major axes, with clinical characteristics: age of onset, body distribution, temporal discrimination and other related features on Axis 1, while etiological factors such as whether the dystonia is acquired or inherited compose Axis 2 (Albanese et al., 2013). Most recent prevalence estimates vary from 20 to 152 cases per million, although these likely to be underestimations due the difficulties and delays in diagnosis (Albanese et al., 2019; Warner et al., 2000; Williams et al., 2017).

Adult-onset idiopathic focal dystonia (AOIFD) is the most prevalent form of dystonia. Cervical dystonia (CD), which affects the muscles of the neck causing abnormal postures of the neck, head and shoulders is the most common (Defazio et al., 2013). Botulinum toxin injections (BTX) into the affected muscles is the first line treatment for people with cervical dystonia (pwCD), which are given at 12-16-week intervals and reduces the activity of the affected muscles (Contarino et al., 2017). Deep brain stimulation (DBS), which typically targets the globus pallidus has also been found to be effective in relieving symptoms for patients with more debilitating and treatment resistant forms of CD. Anticholinergic medications have also been found to be of benefit (Albanese et al., 2019).

Along with other movement disorders, dystonia has traditionally been viewed as a disorder of the basal ganglia. Recent developments in neuroimaging and neurophysiology research have identified the involvement of several other brain regions including the brain stem, the cerebellum, the thalamus, and cortex. It has been suggested that CD would be better conceptualised as a network disorder involving the basal ganglia-thalamo-sensorimotor cortical network (Conte et al., 2020).

Evidence suggests pwCD also experience a spectrum of ‘non-motor symptoms’ that are often prevalent months or years before motor symptom onset. Pain, depression, anxiety, sleep difficulties, sensory abnormalities and cognitive difficulties have been commonly reported and have a greater impact on health-related quality of life (HRQoL), above and beyond the impact of dystonic motor symptoms (Ndukwe et al., 2020; Smit et al., 2016; Yang et al., 2017). PwCD also report significant functional limitations, restrictions in their daily life and increasing levels of disability (van den Dool et al., 2016).

There is increasing interest in the cognitive profile of pwCD. Cognition is a broad term used to describe several related and interdependent mental processes such language, memory, processing speed and executive functions, amongst others. Studies have highlighted a range of subtle cognitive impairments in executive function, attention, working memory and visuospatial function in focal dystonia. However, subgroups of dystonia are frequently studied together (Jahanshahi, 2017; Romano et al., 2014) or cognitive impairments have been considered together with other non-motor symptoms, such as pain or mood difficulties, often with conflicting results across studies (Kuyper et al., 2011; Stamelou et al., 2012). Due to the clinical heterogeneity of the different dystonia subtypes, there have been calls to study different forms of dystonia as separate entities as grouping the subtypes together may mask findings particular to a specific subtype (Conte et al., 2020; Jinnah et al., 2013).

In other movement disorders, such as essential tremor and Parkinson’s disease, cognitive difficulties have been shown to have a significant impact on HRQoL and functional outcomes (Gandy et al., 2018; Louis, 2010); relationships that are largely unexamined in pwCD. Elucidating the cognitive profile of pwCD may have implications for diagnosis and treatment. For health care professionals supporting pwCD, clarifying the cognitive profile will inform assessments and interventions to maximise functioning for pwCD in a holistic and person-centred manner. In addition, if specific cognitive impairments are present, neuropsychological rehabilitation strategies may prove helpful for individuals to work with their strengths, compensate for their weaknesses and to maximise their functioning in everyday life (Wilson et al., 2009). For pwCD, clarifying the cognitive profile may help them develop a coherent understanding of their CD and to make sense of their lived experience, ultimately support their adjustment to living well with CD (Morgan et al., 2019).

To our knowledge, this is the first systematic review to provide an overview of the evidence regarding potential cognitive functions that are affected, or not, in pwCD. Psychological difficulties such as anxiety and depression are highly prevalent in CD (and have recently been reviewed elsewhere, see Girach et al., 2019; Medina Escobar et al., 2021). Given the known impact of psychological difficulties on cognition (Strauss et al., 2006; Zuckerman et al., 2018), the interaction between distress and cognitive outcomes where reported, will also be briefly discussed. The review also aims to discuss the general strengths and limitations of the current literature in addition to recommendations for future research.

## Methods

The current review was conducted in accordance with the Preferred Reporting Items for Systematic Reviews and Meta Analyses (PRISMA) guidelines (Page et al., 2021), Synthesis without meta-analysis (SWiM; Campbell et al., 2020) and was adapted to neuropsychological research as suggested by Gates and March (2016). The review was registered with the International Prospective Register of Systematic Reviews (PROSPERO, ID: CRD42020177720) on 1st of April 2020 and amended on 25th of November 2020 to refine inclusion criteria following preliminary searches.

## Data Sources and Search Strategy

The MEDLINE, PsychINFO, EMBASE, and Web of Science databases were all searched on 5th of May 2020. No time restriction was placed on the literature, but searches were limited to studies of an adult population (> 18 years old) and to those published in English. A broad search strategy was used to capture the recent change in dystonia classification system. The following key terms were used: ‘dystonia’, ‘torticollis’, ‘dystonic disorders’ combined with ‘cognition’ and ‘neuropsychology’. The Medline search string is available in the supplementary material Table S1.

## Inclusion and Exclusion Criteria

The following inclusion criteria were applied: (i) cross-sectional or longitudinal studies of adults pwCD (ii) the results of standardised measures of cognitive or neuropsychological function commonly used in clinical practice (e.g. pen and paper tests) in any form (raw, mean, *z* score, scaled scores, percentiles, standardised scores etc.) were assessed and reported; (iii) the results of the cognitive or neuropsychological tests were compared to a control group or normative data, and (iv) were published in English. Studies reporting on brief cognitive screening measures were included. Case studies, book chapters, abstracts, studies of children under the age of 18 and animal studies were excluded. Mixed AOIFD subgroups, where data could not be differentiated from other forms of dystonia or neurological conditions were also excluded.

## Identification of Relevant Studies and Data Extraction

Searches were completed on 5th of May 2020 and automatic email alerts set to identify any additional studies. The final search was carried out on 5th of April 2021. Reference lists of key papers were also hand searched. Authors of conference proceedings were contacted in the case that some papers may be in press. This resulted in two additional papers for consideration (Ellement et al., 2020; Mahajan et al., 2018). The final database search was completed on 20th of March 2021. Title and abstract screenings were completed by the primary researcher (SOC). All potentially relevant articles were read in full by SOC and TB. Inter-rater reliability for full text screening was high (kappa = 0.85). Data on study and participant demographics and characteristics were extracted (see Table 1). Data on cognitive assessments, significant and not significant results were also extracted (see Table 2). Data extraction and quality assessment was completed independently by SOC, with 35% independently co-rated by SR. There was high inter-rater reliability for data extraction and quality assessment (kappa = 0.97). Conflicts and discrepancies between the reviewers were resolved through discussion until consensus was met.

**Table 1:** Summary and demographics of included studies in chronological order

First author, year	Country	Diagnosis	Control group	*N* (AOIFD, HC)	Mean age (SD)	Gender (F:M)	Treatment/medication
Leplow et al. 1994	Germany	CD	Yes	18 CD 18 HC	CD 51.7 (6.6) HC 52.1 (6.2)	CD 13:5 HC 13:5	33.3% benzodiazepines medication (n = 6)
Hinse et al. 1996	Germany	CD	Yes	15 CD 15 HC	CD 50.9 (23–29) HC 50.4 (30–72)	CD (8:7) HC (8:7)	100% (n = 15) receiving BTX. 6 receiving central active drugs (tri hexyphenidyl (n = 3), amitriptyline (n = 2), flunitrazepam (n = 1), diazepam (n = 1), triapid (n-1)
Rinnerthaler et al. 2006	Austria	CD BSP	Yes	12 BSP 20 CD 32 HC	CD & BSP 54.7 (8.01) HC 54.2 (9.26)	BSP & CD 22:10 HC 22:10	100% (n = 31) receiving BTX
Hoffland et al. 2011	England	CD	Yes	13 CD 29 HC	CD 57.0 (8.6) HC 57.5 (7.0)	CD 9:4 HC 18:11	100% (n = 13) receiving BTX
Sitek et al. 2011^	Poland	CD	Yes	23 HD 25 PDdys 21 PDndys 20 CD	HD 49.83 (11.12) PDdys 65.68 (10.03) PDndys 64.67 (7.59) CD 51.75 (12.98)	HD 9:14 PDdys 13:12 PDndys 6:15 CD 12:8	Not reported
Sitek et al. 2013^	Poland	CD	Yes	23 HD 25 aPD 21 mPD 20 CD	HD 9.83 (11.12) aPD 65.68 (10.3) mPD 64.67 (7.59) CD 51.75 (12.98)	CD12:8	Not reported
Dinkelbach et al. 2015	Germany, Austria	CD	No	13 CD	CD 53.85 (12.16)	7:6	100% (n = 13) Chronic pallidal deep brain stimulation
Chillemi et al. 2018	Italy	CD	Yes	23 CD 12 HC	CD LC 54.83 (8.59) CD TC 54.18 (14.22) HC 52.69 (11.03)	CD 15:8 HC 7:5	100% (n = 23) BTX
Czekóová et al. 2017	Czech Republic	CD	Yes	CD 25 HC 26	CD 56.5 (12.4) HC 56.7 (11.9)	CD 17:8 HC 19:7	100% (n = 25) receiving BTX
Bayram et al. 2018	Turkey	CD	Yes	30 PD 30 CD 30 HC	PD 56.43 (9.21) CD 53.44 (11.2) HC 53.44 (11.2)	PD (10:20) CD (20:10) HC (13:17)	100% (n = 30) PD receiving anti-Parkinson’s medication 100% (n = 30) CD on BTX A
Foley et al. 2017	United Kingdom	CD	Yes	25 CD 13 generalised dystonia 50 HC	CD 57.87 (9.73) Generalised 39.15 (12.79) HC 50.30 (12.49)	CD (19:6) Generalised dystonia (7:6) HC (21:29)	86.84% (n = 33) taking medications at time of testing 30/38 antimuscarinics, 14/38 hypnotics, 2/38 relaxants, 7/38 antidepressants
Yang et al. 2017	China	CD BSP	Yes	60 CD 60 BSP 60 HC	CD 40.23 (13.30) BSP 54.94 (11.51) HC 47 (14.18)	CD 35:25 BSP 38:22 HC 30:30	CD: 17 on anticholinergics or benzodiazepines, 2 BTX, 14 treatment before, 17 no previous treatment BSP: 21 in current treatment, 2 BTX, 10 in treatment before, 17 no previous treatment
Huh et al. 2018	Korea	CD	No	12 CD	CD 56 (35–71)	8:4	DBS GPi Clonazepam and trihexane
Bradnam et al. 2019	Australia	CD	Yes	10 CD 11 HC	CD 50.80 (12.28) HC 43.18 (12.82)	CD 8:2 HC 8:3	70% (n = 7) receiving BTX
Maggi et al. 2019	Italy	CD BSP	Yes	26 CD 27 BSP 30 HC	BSP 65.6 (8.6) CD 60.5 (9.1) HC 61.2 (9.8)	NR	100% (n = 53) receiving BTX 18.52% (n = 5) BSP and 11.54% (n = 2) CD clonazepam
Burke et al. 2020*	Ireland	CD	Yes	46 CD 46 HC	CD 58.79 (10.37) HC 59.86 (5.82)	CD 31:15 HC 32:14	100% (n = 46) receiving BTX
Ellement at al. 2020	Canada	CD	No	46 CD	CD 58.57 (10.84)	40:6	95.65% (n = 44) receiving BTX injections, 19.57% (n = 9) antidepressants,
Herr et al. 2020	Germany	CD	Yes	40 CD 40 HC	CD 61.8 (10.9) HC 61.6 (12.2)	CD 23:17 HC 23:17	75% (n = 30) BTX
Lagravinese et al. 2021	Italy	CD	Yes	35 CD 47 HC	CD 57.63 (13.32) HC 52 (12.66)	CD 17:18 HC 26:21	100% (n = 35) receiving BTX
Monaghan et al. 2021*	Ireland	CD	No	46 CD	68 (10.7)	31:15	100% (n = 46) receiving BTX

Abbreviations: BSP, blepharospasm; CD, cervical dystonia; CD-T, cervical dystonia with tremor; CD-NT, cervical dystonia no tremor; HC, healthy control; HD, Huntington’s disease; HFS, hemifacial spasm; PD, Parkinson’s disease; PDdys, Parkinson’s disease with dyskinesia; PDndys, Parkinson’s disease without dyskinesia; aPD, advanced Parkinson’s disease; mPD, mild Parkinson’s disease; BTX, botulinum toxin treatment; CD LC, cervical dystonia laterocollis; CD TC, cervical dystonia torticollis,

*^ indicate same study cohort

**Table 2:** Summary of results of included studies in chronological order

Study	*N*	Cognitive assessment	Measures with significant differences (AOIFD v HC)	Measures with no significant differences (AOIFD v HC)	NIH QA score	QA comments
Leplow et al. 1994	18 CD 18 HC	Route walking test, body placing test, Ratcliff figures, Benton’s Line Orientation Task, dot localisation, draw-a-bicycle	CD worse than HC: Verbal IQ, route walking test, Draw-a-Bicycle (if Lezak scoring used)	CD v HC: personal orientation test, Ratcliff Figures, Dot Localisation, Benton Line Orientation, draw a bicycle (if Kolb and Wishaw scoring used)	Fair	Some evidence of selection bias Some evidence of information bias Lack of adjustment for multiple comparisons and small *N*
Hinse et al. 1996	15 CD 15 HC	Mehrfachwahl-Wortschatzest (German equivalent of NART), Hebb’s recurring-digits test, Corsi’s block tapping test, Judgement of the visual-vertical, Benton’s line orientation test, Route walking test, personal orientation test, Ratcliff’s mental re-orientation test, standardised road-map of direction sense.	CD worse than HC: Verbal IQ, road map test number of correct turns, body-scheme errors, route walking test correct turns, mental orientation test mean errors	CD v HC: recurring digits test, block tapping test, Line orientation test	Fair	Some evidence of selection bias Some evidence of information bias Lack of adjustment for multiple comparisons and small *N*
Rinnerthaler et al. 2006	12 BSP 20 CD 32 HC	MDRS, VOSP, FEEL	BSP + CD worse than HC: FEEL disgust	BSP + CD v HC: MDRS, VOSP, FEEL total score, FEEL: happiness, fear, surprise, sadness, anger BSP v CD: Feel disgust	Fair	Some evidence of selection bias Lack of statistical power
Hoffland et al. 2011	13 CD 29 HC	NART, A cancellation test of psychomotor speed, graded difficulty arithmetic test, Design Fluency (DKEFS)	CD worse than HC: more errors when copying meaningless gestures for both dominant, non-dominant hand, meaningless sequence with dominant hand, sig more errors on sequence task	CD v HC: NART, A Cancellation test, tapping task, graded difficulty arithmetic test, design fluency CD v HC: no significant differences in copying meaningful gestures with either hand, types of errors, meaningful sequence task with either hand, meaningless sequence performed with the non-dominant hand	Fair	Some selection bias Small *N*, not adjusted for multiple comparisons
Sitek et al. 2011	20 CD 23 HD 25 PDdys 21 PDndys	MMSE, AVLT, Stroop CWIT	CD better than HD: AVLT 1–5, ALVT delayed recall CD better than HD and PDdys: Stroop CWIT	AVLT % recall after a delay	Fair	Some selection and information bias
Sitek et al. 2013	20 CD 23 HD 25 aPD 21 mPD	MMSE, Stroop CWIT	CD better than aPD: Stroop CD less errors than aPD and HD	NR	Fair	Some selection and information bias
Dinkelbach et al. 2015	13 CD	Verbal Learning and Memory Test, DS forward, block span forward, NVLT, DOT, VF(lexical and semantic), TEA (elevator counting, elevator counting with distraction, elevator counting with reversal), Stroop, BJLOT, VOSP, graded difficulty arithmetic test, MWT-A	CD post DBS: sig. worse category fluency	CD post DBS: VLMT, DOT, WMS-R Block span, NVLT, DOT, RWT, TEA, FWIT, VOSP, BJLOT, Verbal IQ (MWT-A)	Good	Small *N*
Chillemi et al. 2018	23 CD 12 HC	Line bisection task	CD worse than HC: line bisection		Fair	Some evidence of selection bias Some evidence of measurement bias
Czekóová et al. 2017	CD 25 HC 26	WAIS-R (picture completion, Similarities, Digit-Symbol Coding, Arithmetic), WMS -III (Word list), Stroop test, TMT, Tower of London, phonemic and semantic VF, FPRT	CD worse than HC: Picture Completion, Arithmetic, Digit-Symbol Coding, TMT A, TMT-B, lexical fluency, semantic fluency, Tower of London, Immediate verbal memory, delayed verbal memory, FPRT +, FPRT + Cognitive inappropriateness, intentions, belief, FPRT + Affective detection, empathy, FPRT -	CD v HC: recognition memory, Stroop test	Fair	Some evidence of selection bias Small *N*, lack of statistical power and multiple comparisons
Bayram et al. 2018	30 PD 30 CD 30 HC	MMSE, VF (phonemic, semantic, action)	CD worse than HC: action verbs	CD v PD v HC: MMSE, Phonemic fluency, Semantic fluency, Action fluency, non-action word counts, action verb counts CD v PD: No between group differences on non-action and action word counts	Fair	Some evidence of selection bias
Foley et al. 2017	25 CD 13 generalised dystonia 50 HC	WAIS-III (prorated verbal and non-verbal abilities), NART, MMSE, Graded Naming Test, VOSP silhouettes/ incomplete letters, Recognition Memory Test, TEA (elevator counting), TMT-A, TMT-B, Modified Card Sorting Test, Brixton Spatial Anticipation Test, Hayling Sentence Completion Test, FAS VF	Combined dystonia worse than HC: TMT-A, TMT-B, TMT-B-TMT-A Combined dystonia: 31.58% (n = 12) impaired on at least of on the TMT-B, Stroop and Hayling tests	CD V generalised: NART, MMSE, Graded naming, VOSP, RMT, TEA, MCST, Brixton, FAS Combined dystonia v HC: NART, WAIS-III, memory	Good	Some evidence of selection bias Lack of statistical power
Yang et al. 2017	60 CD 60 BSP 60 HC	ACE-R	CD + BSP worse than HC: ACE-R total, orientation, memory, fluency, language, visuospatial 42.9% impaired on ACE-R (15 CD, 21 BSP)	CD v BSP: ACE-R total, ACE: orientation, memory, fluency, language, visuospatial	Fair	Some evidence of selection bias Large *N* but no a priori power calculation
Huh et al. 2018	12 CD	The Seoul Neuropsychological Screening Battery: DS forwards and backward, Korean BNT, RCFT (copy, immediate recall, delayed recall, and recognition), Seoul Verbal Learning test (immediate recall, delayed recall, recognition), COWAT, Korean Colour-Word Stroop, Korean MMSE	CD worse post DBS: Stroop word and colour reading correct responses	CD post DBS: DS, BNT, RCFT, Seoul Verbal Learning, COWAT, MMSE	Good	Small *N*
Bradnam et al. 2019	10 CD 11 HC	Line bisection, Bells Task, Landmark Task, Temporal Order Task, Walking Task	CD worse than HC: Walking task. n = 3 with at a neglect like pattern on at least one of the tests (n = 1 Line Bisection Task, n = 2 Temporal Order Task, n = 1 walking task)	CD v HC: Line Bisection, Bells Task, Landmark Task, Temporal Order Task, Walking Task	Fair	Some evidence of selection bias Small *N*, lack of statistical power
Maggi et al. 2019	26 CD 27 BSP	MoCA, MIST, Prose memory test, TMT, MCST	BSP worse than HC: time-based PM, recognition task of MIST CD worse than HC: time-based PM, recognition task of MIST	BSP/CD v HC: MoCA, MCST, TMT, recall of short story,	Fair	Some evidence of selection bias Lack of statistical power
Burke et al. 2020	46 CD 46 HC	TOPF-UK, RAVLT, Logical Memory I and II (WMS-IV), ROCFT, Lexical Fluency, DS, Stroop CWI Test, FAB^, RMET	CD v HC: Name Facial Affect, Name Emotional Prosody, LM encoding, LM delayed recall, LM recognition, LM % retention, RCFT Copy, RCFT immediate, RCFT Delay, RCFT Recognition, RAVLT recognition,	CD v HC: Fluency, Forward Digit Span, Reverse Digit Span, Stroop Colour Naming, Stroop Word Reading, Stroop Trial 3, RMET, Conflicting Emotional Prosody, Matching emotional faces to emotional prosody % retention of RAVLT, % retention of ROCFT	Good	A priori power calculation complete
Ellement et al. 2020	46 CD	Advanced clinical solutions for the WASI-IV & WMS-IV Affect Naming task, Prosody Face Matching task, Prosody Pair Matching task), Benton Facial Recognition Task, reality-known and reality-unknown false belief reasoning test	CD: 21.74% (n = 10) impaired on TOM impaired belief reasoning reality known, 4.35% (n = 2) based on reality-unknown test, 4.35% (n = 2) impaired on both reality known and reality unknown tasks. 10.37% (n = 5) inconclusive results on reality unknown belief reasoning. CD worse than HC: fearful affect naming. CD: sadness, anger, and surprise less well recognised than happiness and disgust, anger, and surprise less well recognised than neutral	Prosody Face Matching, Reality Unknown belief test, reality known belief test	Fair	Some evidence of selection bias Large *N*, although no power calculation
Herr et al. 2020	40 CD 40 HC	MoCA, TMT, DS, FAS VF	CD worse than HC: TMT	CD v HC: MoCA, DST, F-A-S VF	Fair	Some evidence of selection bias Lack of statistical power but large *N* relative to others
Lagravinese et al. 2021	35 CD 47 HC	MMSE, MoCA, Advanced Test of ToM (AT), Emotion Attribution Task (EAT)	CD v HC: MMSE, MoCA total scores	CD-NT v HC: MoCA visuospatial, CD-T v HC: MoCA, orientation CD-T & CD NT v HC: MoCA language, AT, EAT	Fair	Lack of statistical power but large *N* relative to others Controlled for multiple comparisons
Monaghan et al. 2021	46 CD	TOPF, WASI-II, WAIS-IV (digit span, coding), RAVLT, WMS LM, RCFT, BNT, DKEFS phonemic and semantic fluency, Stroop, ^FAB, RMET, Pegboard	CD worse than normative data: Coding, LM I, LM 2, RCFT Immediate, RCFT Delay, RCFT Recognition, FAB naming facial affect, FAB naming emotional prosody, FAB matching, FAB congruent	CD v normative data: TOPF-UK, WASI-II FSIQ, processing speed, BNT, Semantic fluency, RAVLT, RAVLT recall, Digit Span, Stroop Inhibition, letter fluency, RMET, FAB incongruent	Good	Based from previous case-controlled study (Burke et al., 2020) Controlled for multiple comparisons

*Note*: ACE-R = Addenbrooke’s Cognitive Assessment Revised, AOIFD = Adult Onset Idiopathic Focal Dystonia, BNT = Boston Naming Test, BSP = blepharospasm, BTX = Botulinum toxin, CD = cervical dystonia,, COWAT = Controlled Oral Word Association Test, CWIT = Colour Word Interference Test, DBS = Deep Brain Stimulation, D-KEFS = Delis Kaplan Executive Function System, DOT = Digit Ordering Test, DS = Digit Span, DS = Digit Span, FAB = Florida Affect battery, F-A-S = phonemic verbal fluency, FPRT = Faux Pas Recognition Test, HC = healthy controls, HD = Huntington’s disease, HFS = hemifacial spasm, JLOT = Judgement Line Orientation Test, JRS = Jankovic Rating Scale, LM = Logical Memory, MDRS = Mattis Dementia Rating Scale, MCST = Modified Card Sorting Test, MIST = Memory for Intentions Screening Test, MMSE = Mini Mental State Examination, MoCA = Montreal Cognitive Assessment, MWT-A = multiple choice vocabulary test, NART = National Adult Reading Test, NR = Not Reported, NVLT = Non Verbal Learning Test, PD = Parkinson’s disease, PM = Prospective Memory, PST = Prague Stroop Test, RAVLT = Rey Auditory Verbal learning Test, RCFT = Rey-Osterrith Complex Figure Test, RMET = Reading the Mind in the Eyes Test, RWFT = Regensburg’s Word fluency Test, TEA = Test of Everyday Attention, TMT-A = Trail Making Test A, TMT-B = Trail Making Test B, TOPF-UK = Test of Premorbid Function United Kingdom edition, VF = Verbal Fluency, VLMT = Verbal Learning memory Test, VOSP = Visual Object and Space Perception Battery, WAIS = Wechsler Adult Intelligence Scale, WASI = Wechsler Abbreviated Scale of Intelligence, WCST = Wisconsin Card Sorting Test, WMS = Wechsler Memory Scale

## Risk of Bias and Quality of Evidence

The methodological quality and subsequent risk of biases was assessed using the National Heart, Lung and Blood Institute (NHLBI) from the National Institute of Health study quality assessment tools (NHLBI, 2014). Depending on the study design, the observational cohort and cross-sectional, case control and before-after (pre-post) study forms were used. The tools consider the risk of potential selection bias, information bias, measurement bias and other confounding factors through a series of questions, with possible answers as yes, no, other (cannot determine, not applicable or not reported). Questions include: ‘Was a sample size justification, power description, or variance and effect estimates provided?’ and ‘Were key potential confounding variables measured and adjusted statistically for their impact on the relationship between exposure(s) and outcome(s)?’. As per the guidance of the tools, studies were rated as *good, fair*, or *poor* based on the overall qualitative judgement of the scores (i.e., the rating was not rated on average scores or tallies of individual items). The Grading of Recommendations, Assessment, Development and Evaluation (Guyatt et al., 2011) framework was also consulted to consider the quality and strength of the evidence for outcomes (cognitive domains) across studies. According to GRADE, observational studies automatically receive a *low* quality score. Factors that reduce the quality of the evidence include the inconsistency of results, indirectness of the effect, imprecision, risk of bias and publication bias.

**Fig. 1:**
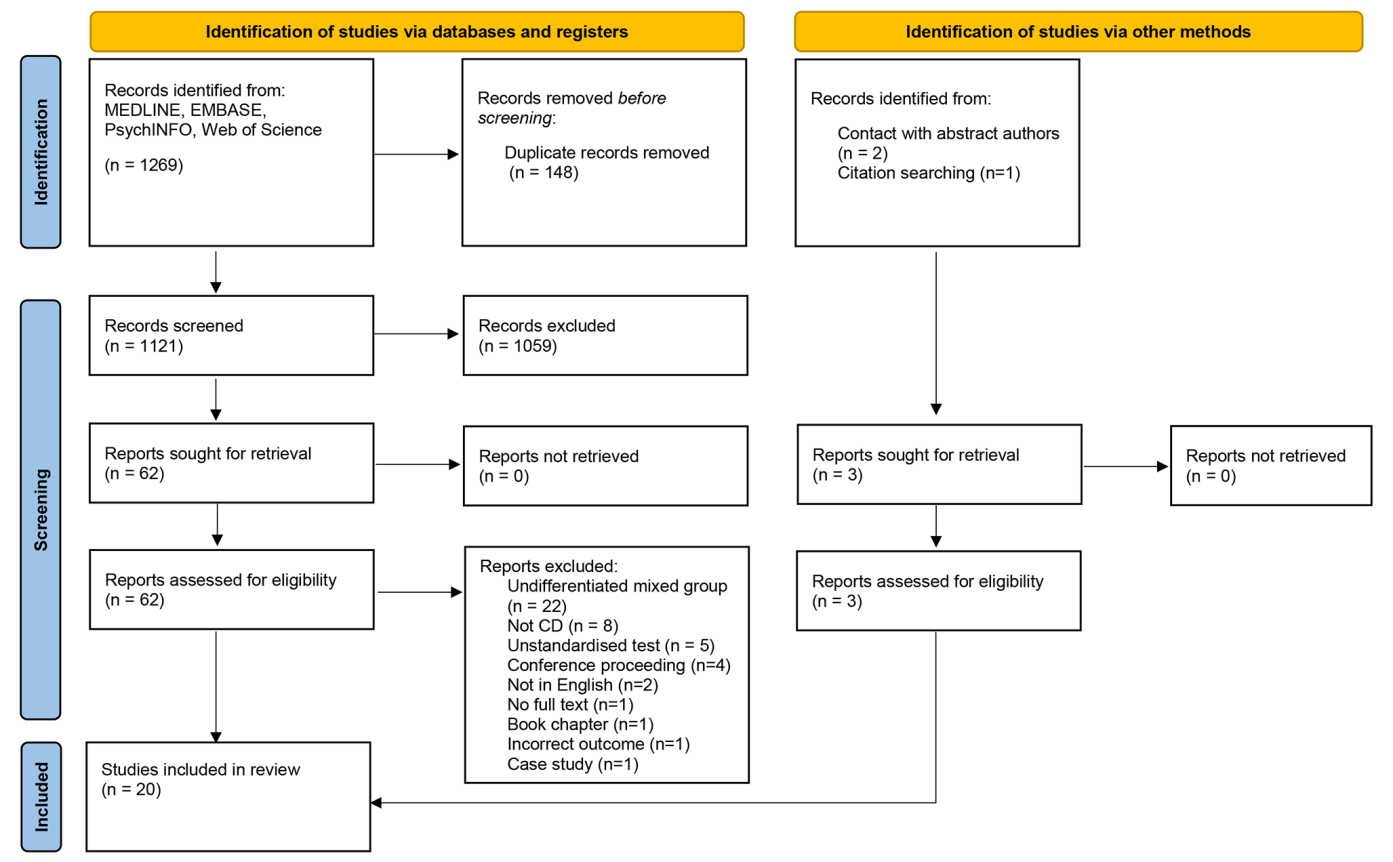
PRISMA diagram depicting flow of studies through the systematic review

## Synthesis

A systematic, narrative synthesis approach was utilised in this study as a quantitative meta-analysis was not deemed appropriate due the diversity in study methodology and outcome data. The assessments, results and conclusions are grouped according to the original authors’ assignment within the study or according to common cognitive categorisations (Lezak et al., 2012), such as general cognition, processing speed, working memory and attention, language, visuospatial functioning, learning and memory and executive functioning if not available within the original study. This is in line with commonly used reporting in clinical neuropsychology and should facilitate ease of reference for clinicians. The allocation of specific cognitive assessments to domains, is available in Supplementary Information Table S2.

## Results

Figure 1 depicts the flow of studies through the phases of the current review. A total of 20 studies met the criteria and were assessed for the review, including 16 case-controlled studies, two cross-sectional studies and two pre-post intervention studies of deep brain stimulation (DBS). The same cohort was presented across Burke et al. (2020) and Monaghan et al. (2021) with a focus on different outcomes. Sitek et al. (2011, 2013) also utilised the same cohort in both papers and reported on different outcomes. For the synthesis, the studies with shared cohorts were merged, and the participants were counted once, with additional outcomes reported as appropriate.

The demographics and characteristics of the included studies are presented in Table 1. The majority of studies came from Europe, with two from Asia, and one each from Canada and Australia. The number of participants were generally small, ranging from 10 to 60. In line with the clinical characteristics of CD, the mean age of the participants varied from early 40’s to 60’s and all papers reported a female gender bias. Sixteen studies reported on cognitive outcomes in pwCD and four studies reported on combined dystonia groups and discussed sub-group results for pwCD. The majority of studies (*n* = 16, 80%) included a control group and two studies reported on cognitive outcomes post DBS. Most studies recruited from outpatient movement disorder clinics. Disease duration was reported in 16 papers and treatment or medication status in 18. Table 2 provides a summary of the results of the studies included in the review.

## General Cognition and Premorbid Function

Measures of general cognitive function typically refers to measures that provide estimates of global cognitive ability or intellectual functioning (IQ). Pre-morbid estimations of ability provide a hypothetical approximation of an individual’s best level of ability, either using education and demographic information or using an individual’s performance on tasks that are thought to be relatively stable to neurological damage (Strauss et al., 2006). Brief cognitive screening batteries such as the Mini Mental State Examination (MMSE) (Folstein et al., 1975) also aim to provide a general overall estimate of an individual’s current cognitive functioning.

Overall, brief screening tools were primarily used as a threshold for inclusion/exclusion in studies of cognitive functioning in pwCD. In the one study that used a brief screening measure to assess cognitive functioning, a quarter of the pwCD were found to experience cognitive impairment. Premorbid function does not appear to be consistently impacted in dystonia. There was mixed support for intact general intellectual functioning, with one study finding no differences between CD, generalised dystonia, and HC, while another study found that 21% of pwCD performed in the impaired range on the WASI-2 (Monaghan et al., 2021). Difficulties in visual perception, quantitative reasoning, and speed of processing were described in another case controlled study, while verbal reasoning was found to be intact (Czekóová et al., 2017).

## Brief Cognitive Screening

Ten studies presented over 11 papers, used general cognitive screening tools such as the MMSE (Folstein et al., 1975) or Montreal Cognitive Assessment (MoCA; Nasreddine et al., 2005) to identify and exclude participants with cognitive impairment and consequently reported no significant differences between dystonia and control groups or normative data (Bayram & Akbostanci, 2018; Dinkelbach et al., 2015; Ellement et al., 2020; Foley et al., 2017; Herr et al., 2020; Huh et al., 2018; Lagravinese et al., 2021; Maggi et al., 2019; Rinnerthaler et al., 2006; Sitek et al., 2011, 2013). Rinnerthaler et al. (2006) excluded one participant from their study based as their score on the MDRS suggested cognitive impairment. Yang et al. (2017) found that 25% (*n* = 15) of pwCD presented with cognitive impairment compared to 35% (*n* = 21) pwBSP and 8% (*n* = 5) of demographically matched health controls (HC). Cognitive impairment was classified as less than 1.5 SD of the HC mean scores (75/100) on the Addenbrooke’s Cognitive Examination-Revised (ACE-R; Mioshi et al., 2006). There were no significant differences between the CD and BSP groups across the subdomain scores or total scores of the ACE-R and when the groups were combined, they had a significantly lower total and subdomain scores relative to HC. Lagravinese and colleagues (2021) found no significant differences on MMSE or MoCA total scores between pwCD and HC. However, when they split the CD group depending on the presence of tremor, they reported that the pwCD-T (with tremor) and pwCD-NT (no tremor) performed more poorly than HC on the language subtask, while pwCD-T and pwCD-NT performed lower than HC on the orientation and visuospatial subtasks respectively.

## Pre-morbid function

Pre-morbid functioning was formally assessed in four studies over five papers. An early study found that pwCD has significantly lower pre-morbid estimates of functioning, as measured by Mehrfachwahl-Wortschatztest (MWT; Lehrl et al., 1995), which subsequently impacted other cognitive scores (Hinse et al., 1996). Pre-morbid functioning was found to be intact and comparable to control groups in two studies in CD (Foley et al., 2017; Hoffland et al., 2011). Premorbid estimates were used to match the CD cohort with HC in another (Burke et al., 2020; Monaghan et al., 2021) further built on this and compared pwCD to normative data and did not report any significant differences on the Test of Premorbid Function cohort. (TOPF-UK; Wechsler, 2011) Premorbid function was also assessed in a pre-post DBS study, and no changes were reported (Dinkelbach et al., 2015).

## General Intellectual Function

Three studies assessed general cognitive functioning using tasks from the Wechsler batteries (Wechsler, 1981; 2008). One study found that pwCD scored significantly lower than HC on the picture completion, arithmetic, and coding subtests, while their performance on the similarities task was comparable (Czekóová et al., 2017). In contrast, another study found that verbal and nonverbal abilities in their mixed group of CD and generalised dystonia did not differ from demographically matched HC (Foley et al., 2017). Although the CD group were found to have higher verbal and nonverbal intelligence quotient (IQ) relative to the generalised dystonia group, the differences were not significant (Foley et al., 2017). 21% of pwCD were found to have abbreviated full-scale intelligence quotient (FSIQ) in the impaired range (*z* < -1.5) as measured by the Wechsler Abbreviated Scale of Intelligence (WASI; Monaghan et al., 2021).

## Motor Function

Motor functioning is a fundamental process in all activities of daily living and is typically assessed using tests of handedness, strength, dexterity, speed, and praxis (Strauss et al., 2006). Relative to HC, pwCD demonstrated intact motor speed, strength and ideomotor skills. Subtle impairments in motor planning and sequencing novel movements, as demonstrated by CD patient’s poorer performance on copying meaningless gestures when compared to HCs were found (Hoffland et al., 2011).

## Sensory and Somatosensory Function

Olfactory and gustatory function was recently assessed in pwCD (Herr et al., 2020). Compared to the demographically matched HC, patients with CD had significantly lower odour threshold, odour identification and taste scores. Regression analyses revealed that age was the main predictor of olfactory decline, lower MoCA scores predicted gustatory decline and pain predicted a lower odour threshold in CD.

## Processing Speed

Processing speed refers to the efficiency with which one can take in visual or verbal information from their environment, integrate it and respond accordingly (Strauss et al., 2006). Overall, the evidence for impaired processing speed in pwCD is inconsistent, with some studies identifying subtle difficulties, while others have found intact performance across a varied set of measures. One case-controlled study found impaired speed of information processing in CD as measured by the Trail Making Test (TMT) and coding tasks (Czekóová et al., 2017). Another study found no significant differences in processing speed between pwCD and HC using the Stroop Colour and Word reading tasks (Burke et al., 2020), however, when the scores were compared to normative data, between 8.7 and 10.8% of the CD scored in the impaired range (Monaghan et al., 2021). Another study found that CD patients performed within the normal range on cancellation tasks, in line with normative data (Hoffland et al., 2011). The TMT-A test was used as a measure of processing speed in two studies. One study found no significant differences between CD and generalised dystonia on the TMT-A; however, when combined, the dystonia group’s scores were significantly lower than the HC group (Foley et al., 2017). Additionally, within the dystonia groups in this study, there were no significant differences on other measures of processing speed as per performance on the Symbol Search and Coding subtests (Wechsler, 1997). Another study found that pwCD performed worse than HC but better when compared to pwBSP on the TMT test, the results did not reach significance after adjusting for multiple comparisons (Maggi et al., 2019).

## Attention and Working Memory

As a cognitive process, attention is comprised of several components. It refers to the capacity to selectively filter, hold and monitor responses to different stimuli and sustain performance on task, while working memory ensures that the information is maintained and available for manipulation (Strauss et al., 2006). In sum, attention and auditory working memory appears to be intact in CD. No significant differences in attention were found between pwCD and generalised dystonia using the Test of Everyday Attention (TEA; Robertson et al., 1994) elevator counting and elevator counting with distraction tests (Foley et al., 2017).

Auditory working memory as assessed by the digit span test was found to be comparable to HC in two case-controlled studies (Burke et al., 2020; Herr et al., 2020) and unimpaired relative to normative data (Monaghan et al., 2021). Two DBS studies did not find any changes in attention or working memory post DBS (Dinkelbach et al., 2015; Huh et al., 2018; Hinse et al., 1996) found that patients performed comparatively to HC on both verbal and visual tests of working memory as measured by recurring digits and block tapping test performances.

## Memory

Memory refers to a complex set of processes that allows a person to encode, store and to later retrieve information (Strauss et al., 2006). There is conflicting and contradictory evidence for memory impairment in CD. Where impairments have been observed, the deficit has been attributed to another cognitive process such as attention and not solely due to a memory deficit, however no attention deficits were found on testing (Burke et al., 2020). Verbal memory was assessed in seven studies over eight papers. PwCD performed significantly better than Huntington’s disease on the list learning trials and recall after a delay; however, performance was comparable across all groups on the percentage recalled after a delay (Sitek et al., 2011). Using a list learning task, one study of pwCD found impaired immediate and delayed verbal memory while recognition was found to be intact relative to the HC group (Czekóová et al., 2017). Recognition memory for words was also found to be intact in another case-controlled study (Foley et al., 2017). In contrast, another case-controlled study employing a list learning task, found no significant differences on the learning, immediate or delayed recall trials and found that recognition memory was impaired (Burke et al., 2020). Significant differences were found on the story memory task, with pwCD performing significantly lower on all trials compared to HC. The authors posit that this discrepancy between the two verbal memory measures likely reflects shallow levels of encoding due to the cognitive load of the story, the lack of repetition of verbal information and an inability to attend to the information for long enough to encode it. The same cohort’s results were also impaired relative to normative data (Monaghan et al., 2021). In contrast, a combined study of pwCD and pwBSP (Maggi et al., 2019) found no significant differences between CD, BSP, and HC groups on the recall of a short story. No change in verbal memory was found in two pre-post DBS studies (Dinkelbach et al., 2015; Huh et al., 2018).

Four studies presented in five papers examined visual memory. Visual memory for faces was found to be comparable in CD, generalised dystonia, and HCs (Foley et al., 2017). CD were found to demonstrate impaired immediate, delayed and recognition memory for the complex figure task relative to HC (Burke et al., 2020), and to normative data (Monaghan et al., 2021). Interestingly, pwCD performance on the copy trial of the figure was also impaired, and performance was independent of mood. The authors concluded that the difficulties observed were a visual representation of the verbal memory-attentional deficits seen during the story memory task (Burke et al., 2020). Two DBS studies again, found no significant differences on visual memory post intervention (Dinkelbach et al., 2015; Huh et al., 2018).

## Visuospatial Functioning

Visuospatial and perceptual function refer to measures that examine object recognition, perception, constructional and spatial abilities. Visual perception difficulties were screened in two studies (Rinnerthaler et al., 2006; Foley et al., 2017), with pwCD found to perform comparably to HC. A facial recognition task was used as a control task of non-emotional expression recognition in another study and all bar one individual passed it (scoring > 39; Ellement et al., 2020). There appears to be conflicting evidence for visual spatial impairments in pwCD, particularly on copying tasks which require a degree of planning and organising and are also sensitive to executive dysfunction (Lezak et al., 2012).

Judgement of spatial perception and orientation in pwCD was found to be intact in two case-controlled studies (Leplow & Stübinger, 1994; Hinse et al., 1996) and post DBS treatment (Dinkelbach et al., 2015) as measured with a line orientation task. Visuospatial neglect, a disorder of visual inattention, was investigated in several studies using a line bisection task. One study found a significant leftward attentional bias in pwCD relative to HC (Chillemi et al., 2018). In contrast, another case-controlled study found no significant between-group differences on several visual neglect measures; however, three pwCD demonstrated a ‘neglect like pattern’ in at least one of the tasks (Bradnam et al., 2019). Spatial orientation was also assessed in two early case-controlled studies. Difficulties with extra personal orientation were found in both studies during a route walking task (Leplow & Stübinger, 1994; Hinse et al., 1996). Conflicting evidence for left-right discrimination was also reported with CD performing comparably to HC on a figures task but significantly impaired on the road map sense of direction task, in addition to body orientation errors (Hinse et al., 1996).

### Visuoconstruction

Visuoconstruction abilities were assessed in three papers. One study found visuoconstruction difficulties during a drawing task when a scoring method emphasising spatial aspects of the drawing was utilised and unimpaired when a more technical method was used (Leplow & Stübinger, 1994). More recently, Burke and colleagues (2020) found that relative to HC, pwCD performed significantly poorer on the copy trial of the complex figure test, independent of pain, symptom severity, or disability levels.

## Language

Language functioning broadly refers to a wide range of cognitive processes including verbal expression, comprehension, and communication (Strauss et al., 2006). Language was examined in two studies using confrontation naming tests only. One study used the naming test as a measure of pre-morbid function to rule out any global cognitive disorder and did not discuss the results in text (Foley et al., 2017). No changes were reported post DBS by Huh and colleagues (2018) on the Korean version of the Boston Naming Test (Ahn et al., 2010).

## Executive Function

Executive function refers to a wide range of cognitive abilities that allow one to effectively perceive, guide and direct behaviour in a flexible and goal directed manner (Lezak et al., 2012; Strauss et al., 2006). The evidence for executive dysfunction in pwCD is mixed. Some studies have found relatively subtle deficits in executive function, including verbal fluency, planning and initiation while response inhibition appears to be relatively intact.

### Response Generation

Phonemic and semantic verbal fluency was found to be broadly intact in CD (Bayram & Akbostanci, 2018; Burke et al., 2020; Herr et al., 2020) and relative to normative data (Monaghan et al., 2021). In contrast, verbal fluency was found to be impaired in another case-controlled study and independent of CD severity (Czekóová et al., 2017). Bayram and Akbostanci (2018) also assessed action fluency in their study, and while there were no significant differences between pwCD and the PD clinical control group and HCs, they found that pwCD produced significantly less action verbs then HCs. Design fluency was found to be intact in another case-controlled study (Hoffland et al., 2011).

### Response Inhibition

The ability to inhibit a dominant response was assessed in five studies presented in seven papers. In two related studies, pwCD were found to perform better than the clinical control groups of Huntington’s disease and advanced Parkinson’s disease (Sitek et al., 2011) and to make fewer errors (Sitek et al., 2013) on the Stroop task. Two case-controlled studies found that inhibition as measured by the Stroop tasks, was relatively intact in pwCD when compared to HC (Burke at al., 2020; Czekóová et al., 2017) and to published norms (Monaghan et al., 2021).

### Planning and Initiation

Planning was assessed using the Tower of London task in one study and found to be impaired in pwCD (Czekóová et al., 2017). Prospective memory (PM) is the process of remembering future planned events and intentions, which provides a measure of self-initiated monitoring. A selective deficit in time-based PM abilities was found in both pwCD and pwBSP when compared to HC. PM recognition was also impaired, which the authors suggested was likely a result of difficulties with cognitive flexibility (Maggi et al., 2019). Interestingly, the difficulties were not identified by the dystonia groups on self-report questionnaires.

### Set Shifting

Shifting abilities, as measured by the card sorting paradigm were found to be intact in CD and BSP relative to HC by Maggi et al. (2019). One study found that 31.6% (*n* = 12) of their combined CD and generalised dystonia group were impaired on at least one measure of executive function (TMT-B, Stroop or Hayling test) relative to HC. The results were independent of age, disease duration, severity of dystonia, mood, and medication status (Foley et al., 2017). The Brixton Spatial Anticipation test (Burgess & Shallice, 1997), a measure of rule detection and rule following, and a modified card sorting test was only administered to the dystonia groups, with the CD group making more errors than the generalised dystonia group, although the results were not significant (Foley et al., 2017).

## Social Cognition

Social cognition refers to a broad set of cognitive processes ranging from more basic emotion recognition skills to inferring the mental state of oneself and others and is essential for social functioning (Frith & Frith, 2012). The evidence suggests that pwCD can present with difficulties with emotion recognition. Difficulties with more complex sociocognitive processes are also evident and the varying results may be related to the assortment of assessments used in the above studies. Different assessments are likely measuring slightly different aspects of ToM such as attributional bias and mentalising, with different cohorts having differing performance on measures of executive function, which is known to relate to mentalising ability. More studies are required to further examine these the meaning and pattern of these difficulties relative to other cognitive outcomes within CD.

### Emotion Recognition

Emotion recognition has been examined in four studies of pwCD. The earliest study to assess emotion recognition found that relative to HC, the combined CD and BSP groups had difficulty recognising disgust, both achieving similar scores (Rinnerthaler et al., 2006). This finding was not replicated in another case-controlled study, which found that disgust was almost always recognised, and the CD cohort instead had significant difficulty identifying fearful facial expressions (Ellement et al., 2020). Sadness, anger, and surprise were also less well recognised than facial expressions of happy and disgust. Methodology and assessment differences likely underpin these differences as in the former study, the facial stimuli were presented briefly (300 ms) in the former study, while in contrast, stimuli were available for up to 10 s in the latter. Ellement and colleagues (2020) also found that better affect naming performance was associated with the presence of comorbid anxiety and the severity of depression, anxiety, and social phobia. They suggested over-processing of emotional stimuli may be a compensatory strategy for increased anxiety. Impairments in emotion recognition were also found relative to HC (Burke et al., 2020) and to normative data (Monaghan et al., 2021), with pwCD demonstrating significant difficulties identifying happy, sad, fearful, angry, and neutral faces across both visual and auditory domains.

### Theory of Mind

Theory of Mind (ToM) was also examined in five studies. Cognitive ToM refers to the ability to understand and reason about the mental state of others, while affective ToM relates to the ability to infer and understand others’ emotional state (Shamay-Tsoory et al., 2007). One study found significant impairments on the Faux Pas Recognition test, which provides a measure of cognitive and affective ToM (Czekóová et al., 2017). Additionally, higher scores on the cognitive ToM subtest were positively related to measures of executive function and semantic verbal fluency, such that better ToM was associated with better performance on working memory and semantic verbal fluency measures. Another study found that 22% of pwCD had impaired belief reasoning compared to normative data using the false belief reasoning tests (Ellement et al., 2020). PwCD were found to have difficulties inferring the mental state of others in two cognitive and affective ToM tasks in another case-controlled study relative to HC (Lagravinese et al., 2021), with pwCD who also had tremor, performing significantly lower than those without tremor. In contrast, ToM as measured with the Reading the Mind in the Eyes Test (RMET; Baron-Cohen et al., 2001) task was found to be intact relative to HC (Burke et al., 2020) and when compared to normative data (Monaghan et al., 2021).

### DBS and cognition in CD

Two studies examined the impact of DBS on cognitive function in CD. Dinkelbach and colleagues (2015) assessed verbal intelligence, memory, executive function, attention, mental arithmetic, visuospatial function in 12 pwCD before and after 12 months of DBS. Except for verbal fluency, cognitive performance was found to be intact 12 months post DBS. Huh and colleagues (2018) also assessed cognitive functioning in 12 pwCD pre and post DBS, with follow up duration ranging from 3 to 11 months. In contrast to the previous study, they found verbal fluency performance, in addition to language, visuospatial, verbal memory, visual memory, and general cognitive screening, to remain stable post DBS. They reported decreased inhibitory control as measured by the Stroop test post DBS. In line with the literature on DBS in dystonia (Jahanshahi et al., 2011), no relationship between the subtle cognitive impairments and CD severity or outcomes were found in either study, with the authors suggesting that DBS appears to be relatively sparing and have minimal functional impact on cognition in CD.

## Psychological distress

Psychological disturbances are frequently reported pwCD. Given the impact of mood on cognitive function (Ahern & Semkovska, 2017), the relationship between distress and cognition was also assessed in the reviewed papers. Psychological factors were assessed in 13 studies in CD. Over half of the studies actively assessed for mood difficulties in conjunction with cognition with 25% (*n* = 5) reporting on the prevalence of distress in their participants. Rates of distress varied, and only one study (Ellement et al., 2020) found that mood had an impact on cognition.

While not reported in every study that assessed for mood difficulties, the rates of clinically significant levels of depression and anxiety varied from 16 to 33% and 26-52.6% respectively (Foley et al., 2017; Yang et al., 2017; Ellement et al., 2020; Burke et al., 2020; Monaghan et al., 2021). Herr and colleagues’ case-controlled study found that pwCD had significantly higher depression and anxiety scores when compared to HC (Herr et al., 2020), although these differences were not controlled for. Another case-controlled study reported that pwCD-NT scored higher than pwCD-T and HC on the BDI, although these results were not significant (Lagravinese et al., 2021). Two studies of combined dystonia groups (CD and generalised dystonia; Foley et al., 2017, BSP and CD; Yang et al., 2017) did not report any significant difference between dystonia group rates of depression and anxiety and that variations in mood were independent of symptom severity and not related to cognitive performance (Foley et al., 2017), further supporting the hypothesis that subtle cognitive deficits are more likely a feature of dystonia.

Three studies used mood measures to screen out participants with any potentially confounding mood difficulties (Rinnerthaler et al., 2006; Czekóová et al., 2017; Maggi et al., 2019) and did not report any between group differences. Two DBS studies did not report any group/mean change in psychological status post DBS (Dinkelbach et al., 2015; Huh et al., 2018). However, the former reported the individual scores, with seven pwCD showing a reduction in BDI-II scores, while five stayed the same or increased in scores (Dinkelbach et al., 2015). Except for Ellement et al. (2020), mood difficulties did not appear to have an impact on cognitive performance, with authors suggesting that the varied levels of cognitive impairment are independent of mood and more likely reflect underlying pathology of CD. PwCD were found to experience less depression in comparison to HD and PD clinical groups (Sitek et al., 2011, 2013).

## Quality Assessment and Strength of the Evidence

The average number of participants was 27 (range 10–60). None of the studies were population based and most studies recruited from a university hospital movement disorder clinic, which is unsurprising given the rarity of CD. The majority of studies used validated measures of disease severity such as the Toronto Western Spasmodic Torticollis Scale (TWSTRS; (Comella et al., 1997), in addition to validated cognitive measures. 63% reported on the prevalence of distress, a potential confounding factor. According to the NIH quality assessment tools, five studies achieved a rating of *good* and fifteen received a *fair* rating. Only one study reported an *a priori* power calculation (Burke et al., 2020). The lack of statistical power in the cohort of studies, in addition to the small number of participants and multiple comparisons in some studies both increases the likelihood of Type 1 and 2 errors, and certainly questions the strength of the conclusions that they present. There was evidence of selection bias across the studies, with a lack sufficient detail into the recruitment and representativeness of the participants. Given the visible nature of CD and the fact that most studies were conducted in outpatient clinics where patients were attending for treatment, blinding of assessors was not possible. Most studies did account for confounding demographic factors such as age, gender, or education in their analysis. It is important to note that there is no current gold standard tool for assessing the quality of an individual study, that there are limitations to the use of different tools and that the quality can be under or overestimated (Mallen et al., 2006; Whiting et al., 2017).

The GRADE criteria were also consulted when considering the strength of the evidence for cognitive impairments across different cognitive domains (Guyatt et al., 2011; Ryan & Hill, 2016). In keeping with the observational nature of the included studies, the certainty of the evidence ranged from *low* to *very low* across all domains. There was some evidence of publication bias for motor function, somatosensory function and language (≤ 2 studies assessed each domain). The high level of inconsistent results across studies also weakened the strength of the evidence for a specific pattern of cognitive and neuropsychological impairments in pwCD. Please see the Supplementary Information for summary tables.

## Discussion

The current review aimed to collate and evaluate the evidence for a profile of cognitive impairments in pwCD, the most common form of AOIFD (Albanese et al., 2019). A total of 20 studies met the inclusion criteria. The evidence for a specific cognitive profile of impairments is inconsistent, with conflicting and mixed results across different studies. Brief cognitive screening measures were the most frequently used assessments and were primarily used to screen out any participants experiencing cognitive impairment. This is another source of potential bias in published studies, as only those who demonstrated good performance on the cognitive screening tools were included.

The results of the review and summary of the evidence thus far appears to suggest that premorbid functioning, attention and working memory appear relatively well preserved in CD. There is mixed evidence for subtle impairments in general intellectual functioning, processing speed, verbal memory, visual memory, visuospatial functioning, executive function, and verbal fluency. There is emerging evidence for impaired social cognition. While the review highlights a growing trend towards using a more comprehensive assessment battery versus isolated assessments, the heterogeneity makes it difficult to compare results across the studies.

The pathophysiology of dystonia is not yet fully understood. Along with other movement disorders, it was initially conceptualised as a disorder of the basal ganglia. The historic view of the basal ganglia as a region of motor control has now expanded. Several distinct yet interconnected direct and indirect frontal-subcortical loops are well established and dysfunction within the circuits have been linked to impairments in memory, spatial attention, executive function, and social cognition in other disorders with basal ganglia involvement such as Parkinson’s Disease (Bodden et al., 2010; Leisman & Melillo, 2013; Tekin & Cummings, 2002). In dystonia, isolated deficits in semantic fluency, set shifting and attention have been postulated to be reflective of executive function deficits arising from dysfunction within these fronto-striatal circuits (Jahanshahi, 2017). Advances in functional and structural neuroimaging have identified brain changes in other regions and have been offered as possible explanations into the diverse range of cognitive changes seen in dystonia. Increasing attention is being paid to the cerebellum, another region historically linked to motor function. The cerebellum has multiple cortical connections to the thalamus and the prefrontal cortex and neuropsychological studies in healthy controls and in clinical populations suggest that dysfunction in the cerebellum itself or its networks are associated with visuospatial, attention, language, memory and executive function deficits (O’Halloran et al., 2012) and with deficits in social cognition (Van Overwalle et al., 2020). Cognitive deficits in visual and verbal attention, working memory, verbal fluency, verbal memory, and executive function have been identified in essential tremor, a movement disorder with known cerebellar involvement and dysfunction within the cortical-subcortical-cerebellar loops (Bermejo-Pareja & Puertas-Martín, 2012), which also point to the role of cerebellar dysfunction in to the varied cognitive impairments described in CD. Functional connectivity and metabolism changes in the cerebellum have been found in different forms of dystonia (Shakkottai et al., 2017) and in CD, reduced cerebellum activation and subsequent reduced connectivity to the basal ganglia and motor cortex networks was found during a visuospatial task (Filip et al., 2017). Altered temporal discrimination thresholds corresponding to disruptions to the collicular-pulvinar-amygdala network have also been identified in dystonia and offered as a possible explanation for the subtle cognitive and social cognitive deficits seen in CD (Conte et al., 2017; Burke et al., 2020). There are also a range of inconsistencies in neuroimaging studies across dystonia subtypes, which have been suggested to reflect the heterogeneity in clinical manifestations (Lehéricy et al., 2013).

Descriptions of the associations between the various brain regions implicated in CD to the range of cognitive impairments are limited by the lack of studies that combine cognitive and neuropsychological assessments with brain imaging studies. None of the studies included in the review carried out any concurrent neuroimaging. However, the majority of authors did attempt to associate their findings with recent imaging and physiological studies, acknowledging the current view that dystonia should be conceptualised as a network disorder, that involves altered communication in the striato-thalamo-cortical and cerebellar-thalamo-cortical networks as opposed to locating dysfunction to a single site (Conte et al., 2020). Many studies in the current review related their findings to altered connectivity or to the distal effects of dysfunction in a node such as the basal ganglia or cerebellum in these networks. While structural and connectivity changes are well documented in dystonia, the underlying cause of these changes remains unknown.

## Limitations of the Evidence in the Review

The results of this review must be interpreted in the context of the limitations of the current literature and the review process. This review aimed to draw conclusions about domains of cognitive impairment in CD. However, the overall quality of the evidence identified was low, attributable to likely underpowered studies, with a range of methodological issues and biases (Button et al., 2013). Pre-morbid functioning was not assessed in every study, which makes it difficult to ascribe any impairments found as a direct consequence of CD. A wide range of cognitive assessments were used across the studies, which although not surprising and likely to reflect variations in clinical practice, makes it difficult to compare results across studies and may account for some of the inconsistent results seen within cognitive domains. Authors occasionally differed in their interpretation of different cognitive assessments and associated domains (e.g., TMT as a measure of visual attention or speed of information processing). It is widely acknowledged that ‘pure’ measures of cognitive function are rare, and assessments likely measure a range of cognitive abilities (Lezak et al., 2012). This is particularly relevant when considering multifactorial domains such as attention and executive function. Potentially confounding factors such as mood, co-morbidities, pain, and medications were not consistently measured or controlled for in all of the included studies. In many of the studies included, pwCD were also prescribed anticholinergics and benzodiazepines in addition to receiving BTX treatment for their symptoms, which have been shown to have an impact on cognitive function (Federico et al., 2017). Other potential confounding factors such as depression, anxiety or other common psychiatric co-morbidities were not adequately controlled for, which is an important consideration for future studies given the known impact of mood difficulties on cognition in addition to the effect of psychiatric medications (Prado et al., 2018). Depending on where they are in the BTX treatment cycle at the time of the cognitive assessment, pwCD may experience the emergence of motor symptoms and pain and attempts to control them during assessments may impact on their performance (Jahanshahi et al., 2003). Several studies screened out any pwCD who presented with cognitive impairment or mood difficulties, another source of potential bias. An additional limitation of the current literature is the lack of longitudinal studies which preclude any conclusions about whether subtle cognitive impairments where present, remain static or are likely to develop and change over time.

## Limitations of the Review Process

While the current review was conducted systematically and in line with best practice guidelines (Page et al., 2021), it is not without its limitations. The heterogeneity of the included studies meant that a meta-analysis was not possible. For pragmatic reasons, this review was limited to peer reviewed studies published in English, an inherent publication bias in this review (Ioannidis et al., 2014). A strength of the current review was the broad search strategy and the quality assessment of the included studies. Due to the strict inclusion criteria, studies that assessed pwCD with other forms of dystonia who did not analyse subsets were excluded. Studies that used experimental measures of cognition were also excluded but noted difficulties have been identified using e.g. n-Back and CANTAB measures (Romano et al., 2014; Scott et al., 2003). Additionally, the initial search did not identify any research of cognitive outcomes in other forms of AOIFD such as focal hand dystonia (FHD), oromandibular dystonia (OMD) or spasmodic dystonia (SD) which also need to be addressed in future research.

The classification of assessments to different domains was made to reflect consensus in clinical neuropsychology practice and the wider literature base. However, it is acknowledged that different assessments may be classified in different ways (Harvey, 2019; Lezak et al., 2012). To that end, efforts were made to describe the tasks used in each study so that readers may interpret them according to their orientation. According to GRADE, there was a publication bias for the domains of motor function, somatosensory function, and language as they were assessed in fewer than two studies and these results should be interpreted with caution.

## Implications for Research

The results of this review and the methodological limitations discussed, suggest several avenues for future research. Adequately powered studies need to clarify and build on the inconsistent results outlined using a comprehensive and validated range of assessments that are sufficiently sensitive to capture a range of subtle impairments, as even seemingly small impairments can have a range of significant functional consequences. Participants who present with difficulties on cognitive and mood screening measures should also be included and assessed as a separate group to gain a true understanding of the range of impairments in CD. While there is no recommended test battery for pwCD, the range of assessments should be broad enough to capture several cognitive domains. Researchers are encouraged to report effect sizes and confidence intervals so that a more thorough analysis can be carried out in future.

As discussed above, the contribution of other confounding factors such as pain, medications and motor symptoms need to be controlled for in future research. As understanding into the pathophysiology of CD continues to emerge, studies combining neuropsychological assessments concurrently with neuroimaging will clarify the relationships between the neural substrates implicated in CD and the association to cognitive functions. The relationship between severity of dystonia and cognitive functioning also remains to be investigated in more detail. With the issues described above in mind, large, multicentre studies are recommended for several reasons. First, it will allow for sharing of knowledge and resources across specialist dystonia centres. Second, given the relative rarity of CD compared to other movement disorders, it will allow for significantly larger and adequately powered studies that will increase confidence in the findings of future research. The development and agreement of a pre-specified cognitive and neuropsychological testing battery across study sites will also address some of the inconsistencies described in the current literature. Larger studies will also allow more in-depth studies into studies of subgroups of CD, for example, facilitating comparisons of cognitive performance between pwCD with or without mood difficulties or between those who do or do not experience cognitive impairments. Sub-groups of pwCD may emerge in terms of cognitive difficulties which may help clarify the inconsistent findings when looking at larger group means.

While outside the scope of the current review, measuring additional outcomes such as HRQoL, in addition to psychological difficulties need to be assessed in tandem with cognitive measures to further our understanding into the impact of these difficulties for pwCD. Even subtle cognitive impairments have the potential to have a great impact on day to day functioning. PwCD are reported to experience high levels of functional disability and reduced HRQoL (De Pauw et al., 2017; Stamelou et al., 2012) and while the impact of non-motor symptoms is well recognised the relationship between cognitive impairment and daily functioning and other outcomes remains to be investigated. It would also be important to develop understanding into the subjective perceptions of any cognitive impairments in pwCD, and to that end, the inclusion of self-report measures of cognitive function would be important for future studies.

## Implications for Practice

Clinicians should be aware that some pwCD may present with subtle cognitive impairments and evaluate their needs on a case by case basis. A range of cognitive processes work together to facilitate us to engage and participate in our everyday life and even subtle impairments can have a significant impact on functioning. In many cases, the impact of cognitive difficulties can be compensated for with specific strategies tailored to the individual (Wilson et al., 2009). Higher order cognitive functions such as social cognition, which enables us to process emotional stimuli and infer the mental state of others around us can have a greater impact on interpersonal relationships, social functioning and quality of life than other cognitive functions (Fett et al., 2011). Given the high levels of psychological distress, isolation, disability, and stigma reported in pwCD (De Pauw et al., 2017; Kuyper et al., 2011; Morgan et al., 2019); the emerging evidence for social cognition difficulties in pwCD suggests that this area of functioning should be examined in further detail and worth consideration from a clinical perspective.

Examining the cognitive profile of CD in more detail will facilitate the shift away from the medicalised management of the motor symptoms to a more holistic, biopsychosocial understanding of the impact of AOIFD (Wade & Halligan, 2017). Neuropsychological assessment can aid the identification of difficulties in addition to predicting and supporting functional outcomes (Donders, 2020), particularly relevant where difficulties may be subtle. Clinical psychologists, neurologists and other healthcare professionals working therapeutically with pwCD should be alert to the possibility that some pwCD may experience subtle cognitive impairments and tailor person-centred assessments, biopsychosocial formulations, and therapeutic interventions appropriately to account for such difficulties in collaboration with the individual. For pwCD, clarifying and investigating any cognitive difficulties may help them make sense of their experience, adjust to living with CD and develop a coherent identity (Morgan et al., 2019). While the evidence base for cognitive rehabilitation in CD is lacking, there is emerging evidence suggesting that cognitive behaviour therapy and mindfulness may be effective psychological interventions for pwCD (Kobayashi et al., 2020; Sandhu et al., 2016; Wadon et al., 2020). As in other movement disorders with a known profile of cognitive impairments and significant psychological comorbidities, adaptations to psychological interventions may prove beneficial (British Psychological Society, 2021).

## Conclusion

The evidence for specific cognitive impairments in CD is gradually emerging, however the reliability of our current conclusions is limited by the significant short comings of the existing literature. These include a lack of prospective cohort studies, small sample sizes as well as measurement issues within studies. While dystonia is one of the most common movement disorders, there are frequent delays to diagnosis (Defazio et al., 2013). Due to its relative rarity, it is not unsurprising that studies are small and that inconsistent results are seen across studies (Whicher et al., 2018). PwCD may experience subtle difficulties with processing speed, verbal memory, visual memory, visuospatial function, executive function, and social cognition while general intellectual functioning and attention and working memory appear to be unaffected. Methodological issues and inconsistencies across studies question the strength of the evidence provided and future studies need to adequately account for these issues before firm conclusions can be drawn. Adequately powered, multicentre studies will allow further in-depth examination of the prevalence and severity of the cognitive profile, and if combined with neuroimaging, increase our understanding into the cause and consequences of CD.

### Electronic Supplementary Material

Below is the link to the electronic supplementary material.

Supplementary Material 1

## Data Availability

Not applicable.
