# Supplementary material for: A Systematic Review of Cognition in Cervical Dystonia
Source: Neuropsychol Rev. 2023 Jan 25;34(1):134–54. doi: 10.1007/s11065-022-09558-z (PMC10920436; doi:10.1007/s11065-022-09558-z)
Supplement: Supplementary file 1 — Supplementary Material 1 [file 11065_2022_9558_MOESM1_ESM.docx]

**Supplementary Information**

**Table S1**

*Search terms for Medline database*

| Search | Terms |
| --- | --- |
| 1 | (MM "Dystonia+") OR "dystonia" OR (MM "Torticollis") OR (MM "Dystonic Disorders+") |
| 2 | (MM "Torticollis") OR "cervical dystonia" |
| 3 | (MM "Cognition+") OR "cognition" OR (MM "Cognition Disorders+") OR (MM "Social Cognition") |
| 4 | (MM "Cognitive Dysfunction+") |
| 5 | (MM "Neuropsychology") OR "neuropsychology" OR (MM "Neuropsychological Test+") |
| 6 | S1 OR S2 |
| 7 | S3 OR S4 OR S5 |
| 8 | S6 AND S7 |
| 9  10 | Narrow by Language: - english  Narrow by SubjectAge:- all adult: 19+years |

| **Table S2**  *Allocation of Cognitive Assessments to Cognitive Domains* | |
| --- | --- |
| Cognitive Test | References |
| Cognitive screening measures and cognitive batteries | |
| Mini Mental State Examination (MMSE)  Montreal Cognitive Assessment (MoCA)  Mattis Dementia Rating Scale (MDRS)  Addenbrook’s Cognitive Examination-Revised (ACE-R)  Seoul Neuropsychological Screening Battery | Folstein, M. F., Folstein, S. E., & McHugh, P. R. (1975)  Nasreddine, Z. S., Phillips, N. A., Bédirian, V., Charbonneau, S., Whitehead, V., Collin, I., ... & Chertkow, H. (2005)  Mattis, S. (1988)  Mioshi, E., Dawson, K., Mitchell, J., Arnold, R., & Hodges, J. R. (2006)  Ahn, H. J., Chin, J., Park, A., Lee, B. H., Suh, M. K., Seo, S. W., & Na, D. L. (2010) |
| Current General Intellectual Functioning | |
| Wechsler Adult Intelligence Scales (WAIS) | Wechsler D. (1997; 2008) |
| Pre-morbid functioning | |
| Test of Pre-Morbid Functioning United Kingdom (TOPF-UK)  National Adult Reading Test (NART)  Multiple choice vocabulary test | Wechsler, D. (2011)  Nelson H.,E. (1982)  Lehrl, S., Triebig, G., & Fischer. (1995) |
| Attention and Working Memory | |
| Digit Span (WAIS)  Block Span (WMS-R)  Test of Everyday Attention (TEA)  Toulouse-Pieron Test  Graded mental arithmetic  Corsi’s block tapping test | Robertson, I. H., Ward, T., Ridgeway, V., Nimmo-Smith, I. (1996)  Toulouse, E., & Pieron, H. (1986)  Kessels, R. P., Van Zandvoort, M. J., Postma, A., Kappelle, L. J., & De Haan, E. H. (2000)..  Jackson, M., Warrington, E. K. (1986) |
| Processing Speed | |
| Symbol-Digit Coding (WAIS)  Trail Making Test A (TMT A)  A cancellation test | Reitan, R. M., Wolfson, D. (1985)  Willison, J. R., & Warrington, E. K. (1992) |
| Verbal Memory | |
| Auditory Verbal Learning Test (RAVLT)  List Learning Wechsler Memory Scales (WMS)  Recognition Memory Test  Logical Memory Wechsler Memory Scales  Prose story memory  Memory for Intentions Screening Test (MCST) | Spreen, O., & Strauss, E. (1998)  Warrington, E. K. (1984)  Wechsler, D. (1987;2009)  Raskin, S. (2004). |
| Visuospatial Memory | |
| Rey Complex Figure Test (RCFT)  Spatial Location (WMS)  Benton Facial Recognition test | Benton, A. L., Sivan, A. B., Hamsher, K. deS., Varney, N. R., & Spreen, O. (1994)  Wechsler, D. (1987)  Meyers, J.E. and Meyers, K.R. (1995) |
| Visuospatial function | |
| Visual Object and Space Perception battery (VOSP)  Line bisection  Benton Judgement of Line Orientation Test  Draw-a-bicycle | Warrington, E. K., & James M. (1991).  Benton, A. L., Varney, N. R., & Hamsher, K. S. (1978)  Benton, A. L., Sivan, A. B., Hamsher, K. Des., Varney, N. R., & Spreen, O. (1994).  Lezak, M. D. (1983) |
| Language | |
| Graded Naming Test (GNT)  Korean Boston Naming Test (K-BNT) | McKenna, P., & Warrington, E. K. (1983). |
| Executive Function | |
| Verbal Fluency (VF)  Design Fluency  Card Sorting Test  Tower of London  Stroop Colour Word Interference test  Five Digits Test  Hayling Sentence Completion  Brixton Spatial Anticipation Test  Trail Making Test B (TMT B)  Luria’s task | Nelson, H. (1976)  Trenerry, M. R., Crosson, B., DeBoe, J., et al. (1989)  Burgess, P.W., Shallice, T. (1997)  Reitan, R. M., & Wolfson, D. (1985)  Delis, D. C., Kaplan, E., & Kramer, J. H. (2001)  Heaton, R. K., Chelune, G.J., Talley, J. L., Kay, G. G., Curtiss, G. (1993)  Roberts, K. L., & Hall, D. A. (2008)  Bhimani, A.A., Hlustik, P., Small, S. L, Solodkin, A. (2006)  Heaton, R. K., Chelune, G. J., Talley, J. L., Kay, G. G., Curtiss, G. (1993)  Lezak, M. D. (1983) |
| Social Cognition | |
| Reading the Mind in the Eyes (RMET)  Florida Affect Battery (FAB)  Affect naming  Prosody Face Matching Task  Benton Facial Recognition  Reality-known and reality unknown false belief test  Faux Pas Recognition Test  Advanced Test of ToM  Emotion Attribution Task | Baron‐Cohen, S., Wheelwright, S., Hill, J., Raste, Y., & Plumb, I. (2001)  Bowers, D., Blonder, L. X., & Heilman, K. M. (1998)  Benton, A. L., Sivan, A. B., Hamsher, K. deS., Varney, N. R., & Spreen, O. (1994)  Gweon, H., Saxe, R. (2013)  Stone, V. E., Baron-Cohen, S. & Knight, R.T. (1998)  Prior, M., Sartori, G., & Marchi, S. (2003) |
| Motor Function | |
| Perdue Pegboard  finger tapping test | Spreen, O., & Strauss, E. (1998) |
| Somatosensory, Olfactory and body orientation and perception | |
| Route walking  Ratcliff’s Mental Re-orientation Test  Sniffin Sticks  Taste Strips | Semmes, J., Weinstein, S., Ghent, L., & Teuber, H. L. (1963)  Ratcliff, G. (1979)  Hummel, T., Kobal, G., Gudziol, M., Mackay-Sim, A. (2007)  Mueller, C., Kallert, S., Renner, B., Stiassny, K., Temmel, A. F., Hummel, T., Kobal, G. (2003) |

| **Table S3** | | | | | | | | |
| --- | --- | --- | --- | --- | --- | --- | --- | --- |
| *Grading of Recommendations, Assessment, Development and Evaluation (GRADE): Strength and Certainty of Evidence* | | | | | | | | |
| Outcome | No of studies | Study design | Risk of bias | Inconsistency | Indirectness | Imprecision | Publication bias | Certainty |
| Brief cognitive screening | 10 | Observational | Moderate | Not serious | Not serious | Not serious | Unclear | Low |
| Pre-morbid function | 5 | Observational | Moderate | Not serious | Not serious | Serious | Unclear | Low |
| General intellectual function | 3 | Observational | Moderate | Not serious | Not serious | Serious | Unclear | Low |
| Motor function | 2 | Observational | Moderate | Not serious | Not serious | Serious | Strongly suspected | Low |
| Somatosensory | 1 | Observational | Moderate | Serious | Not serious | Serious | Strongly suspected | Very low |
| Processing speed | 5 | Observational | Moderate | Serious | Not serious | Not serious | Unclear | Very low |
| Attention/working memory | 6 | Observational | Moderate | Not serious | Not serious | Not serious | Unclear | Low |
| Verbal memory | 7 | Observational | Moderate | Serious | Not serious | Not serious | Unclear | Very low |
| Visual memory | 4 | Observational | Moderate | Serious | Not serious | Not serious | Unclear | Very low |
| Visuospatial function | 8 | Observational | Moderate | Serious | Not serious | Serious | Unclear | Very low |
| Visuoconstruction | 4 | Observational  Longitudinal | Moderate | Serious | Not serious | Serious | Unclear | Very low |
| Language | 2 | Observational | Moderate | Serious | Not serious | Serious | Strong suspected | Very low |
| Response generation | 7 | Observational | Moderate | Serious | Not serious | Not serious | Unclear | Very low |
| Response inhibition | 5 | Observational | Moderate | Serious | Not serious | Not serious | Unclear | Very low |
| Planning/initiation | 2 | Observational | Moderate | Serious | Not serious | Serious | Strongly suspected | Very low |
| Set shifting | 2 | Observational | Moderate | Not serious | Not serious | Not serious | Unclear | Low |
| Emotion recognition | 4 | Observational | Moderate | Not serious | Not serious | Not serious | Unclear | Low |
| Theory of Mind | 5 | Observational | Moderate | Serious | Not serious | Not serious | Unclear | Very low |

**Table S4**

*Quality assessment of included studies according to the NHLBI Quality Assessment Tools*

| Case-controlled studies (*n*=16) | | | | | | | | | | | | | | | | | | | | | | | | | | | | | | | | | | | | | | | | | |  |  |
| --- | --- | --- | --- | --- | --- | --- | --- | --- | --- | --- | --- | --- | --- | --- | --- | --- | --- | --- | --- | --- | --- | --- | --- | --- | --- | --- | --- | --- | --- | --- | --- | --- | --- | --- | --- | --- | --- | --- | --- | --- | --- | --- | --- |
| Study ID | 1. Research question | | 2. Study population | | 3. Target population and representation | | 4. Sample size | | | 5. Group recruitment | | | 6. Inclusion/exclusion criteria | | | 7. Cases and control definition | | | 8. Random selection | | | 9. Concurrent controls | | | 10. Exposure | | | 11. Exposure measures and assessment | | | 12. Blinding of assessors | | | 13. Statistical analysis | | |  | | | Quality Rating | | |  |
| Hinse 1996 | Y | | NR | | NR | | N | | | NR | | | Y | | | Y | | | NR | | | NR | | | Y | | | Y | | | N | | | Y | | |  | | | F | | |  |
| Leplow 1994 | Y | | NR | | NR | | N | | | Y | | | Y | | | Y | | | NR | | | NR | | | Y | | | Y | | | N | | | N | | |  | | | F | | |  |
| Hoffland 2011 | Y | | NR | | NR | | N | | | NR | | | Y | | | Y | | | N | | | N | | | Y | | | Y | | | N | | | Y | | |  | | | F | | |  |
| Sitek 2011 | Y | | Y | | NR | | N | | | Y | | | Y | | | Y | | | N | | | NR | | | Y | | | Y | | | N | | | N | | |  | | | F | | |  |
| Sitek 2013 | Y | | Y | | NR | | N | | | Y | | | Y | | | Y | | | NR | | | N | | | Y | | | Y | | | N | | | N | | |  | | | F | | |  |
| Czekóová  2017 | Y | | NR | | NR | | N | | | Y | | | Y | | | Y | | | NR | | | NR | | | Y | | | Y | | | N | | | Y | | |  | | | F | | |  |
| Bayram 2018 | Y | | NR | | NR | | N | | | NR | | | Y | | | Y | | | NR | | | NR | | | Y | | | Y | | | N | | | Y | | |  | | | F | | |  |
| Chillemi 2018 | Y | | NR | | NR | | N | | | Y | | | NR | | | Y | | | NR | | | NR | | | Y | | | Y | | | N | | | Y | | |  | | | F | | |  |
| Bradnam 2019 | Y | | NR | | NR | | N | | | NR | | | NR | | | Y | | | NR | | | NR | | | Y | | | Y | | | N | | | Y | | |  | | | F | | |  |
| Burke 2020 | Y | | Y | | NR | | Y | | | Y | | | Y | | | Y | | | N | | | NA | | | Y | | | Y | | | N | | | Y | | |  | | | G | | |  |
| Herr 2020 | Y | | NR | | NR | | N | | | Y | | | Y | | | Y | | | NR | | | NR | | | Y | | | Y | | | N | | | Y | | |  | | | F | | |  |
| Lagravinese 2021 | Y | | NR | | NR | | N | | | NR | | | Y | | | Y | | | NR | | | NR | | | Y | | | Y | | | N | | | Y | | |  | | | F | | |  |
| Rinnerthaler 2006 | Y | | NR | | NR | | N | | | NR | | | Y | | | Y | | | NR | | | NR | | | Y | | | Y | | | N | | | Y | | |  | | | F | | |  |
| Foley 2017 | Y | | Y | | NR | | N | | | Y | | | Y | | | Y | | | N | | | NA | | | Y | | | Y | | | N | | | Y | | |  | | | G | | |  |
| Yang 2017 | Y | | Y | | NR | | N | | | Y | | | CD | | | Y | | | NR | | | NR | | | Y | | | Y | | | N | | | Y | | |  | | | F | | |  |
| Maggi 2019 | Y | | NR | | NR | | N | | | Y | | | Y | | | Y | | | N | | | NR | | | Y | | | Y | | | N | | | Y | | |  | | | F | | |  |
| Observational and cohort studies (*n*=2) | | | | | | | | |  | | |  | | |  | | |  | | |  | | |  | | |  | | |  | | |  | | |  | | |  | | |  |  |
| Study ID | 1. Research question | | 2. Study population | | 3. Participation rate | | 4. Group recruitment | | | 5. Sample size justification | | | 6. Exposure assessed | | | 7. Sufficient time frame | | | 8. Levels of exposure | | | 9. Exposure measures and assessment | | | 10. Repeated exposure measurement | | | 11. Outcome measurement | | | 12. Blinding of outcome assessors | | | 13. Follow-up rate | | | 14. Statistical analysis | | | Quality rating | | |  |
| Ellement 2019 | Y | | NR | | NR | | Y | | | N | | | Y | | | Y | | | NA | | | Y | | | NA | | | Y | | | N | | | NA | | | Y | | | F | | |  |
| Monaghan 2021 | Y | | Y | | NR | | Y | | | N | | | Y | | | Y | | | NA | | | Y | | | NA | | | Y | | | N | | | NA | | | Y | | | G | | |  |
| Pre-post studies with no control group (*n*=2) | | | | | | | | |  | | |  | | |  | | |  | | |  | | |  | | |  | | |  | | |  | | |  | | |  | | |  |  |
| Study | | 1. Study question | | 2. Eligibility and study population | | 3. Representative of population | | 4. All eligible enrolled | | | 5. Sample size sufficient | | | 6. Intervention described | | | 7. Valid outcome measurement | | | 8. Blinding | | | 9. Follow-up rate | | | 10. Statistical analysis | | | 11. Multiple outcome measures | | | 12. Group and individual outcomes | | |  | | |  | | | Quality Rating | | |
| Dinkelbach 2015 | | Y | | Y | | Y | | Y | | | NA | | | Y | | | Y | | | N | | | Y | | | Y | | | N | | | Y | | |  | | |  | | | G | | |
| Huh 2018 | | Y | | Y | | Y | | Y | | | NA | | | Y | | | Y | | | N | | | Y | | | Y | | | N | | | Y | | |  | | |  | | | G | | |
| *Note*. Y, yes; N, no; CD, cannot determine; NA, not applicable; NR, not reported; G, good; F, fair; P, poor. | | | | | | | | | | | | | | | | | | | | | | | | | | | | | | | | | | | |  | | |  | | |  |  |
